# Future planning for athletes: a social cognitive career theory explanation of student-athletes’ self-efficacy and reflection

**DOI:** 10.3389/fpsyg.2026.1828527

**Published:** 2026-06-12

**Authors:** Hsin Chuan Chen, Hsueh-Chung Wang

**Affiliations:** 1 Bachelor Program of International Sport Affairs, National Taiwan University, Taipei, Taiwan; 2 Office of Physical Education, Ming Chi University of Technology, New Taipei, Taiwan

**Keywords:** student-athletes, student identity, general self-efficacy, planning horizon, reflected appraisal, social cognitive career theory, dual career

## Abstract

**Introduction:**

Student-athletes often develop within dual-career contexts in which academic and athletic demands coexist under sustained pressure. Although Social Cognitive Career Theory has been widely used to explain educational and career development, less is known about how identity-based self-systems are translated into extended future-oriented planning under chronic role overload. The study examined whether general self-efficacy mediates the relationship between student identity and planning horizon, and whether reflected appraisal strengthens this pathway among student-athletes.

**Methods:**

A cross-sectional survey design was adopted. Data were collected from 397 intercollegiate student-athletes in Taiwan. Measures included student identity, general self-efficacy, planning horizon, and reflected appraisal. Gender, age, and years of sport-specific training were treated as control variables. Descriptive statistics and Pearson’s correlations were first conducted, and the mediation and moderated mediation analyses were then tested.

**Results:**

Student identity was positively associated with general self-efficacy and planning horizon. General self-efficacy was also positively associated with planning horizon. Mediation analysis showed that general self-efficacy mediated the relationship between student identity and planning horizon. In addition, reflected appraisal significantly moderated the relationship between student identity and general self-efficacy, such that the positive association became stronger as reflected appraisal increased. The moderated mediation analysis further indicated that the indirect effect of student identity on planning horizon through general self-efficacy was conditional on reflected appraisal and became stronger at higher levels of reflected appraisal.

**Conclusion:**

The findings support a Social Cognitive Career Theory-based explanation of student-athletes’ future-oriented planning by showing that student identity contributes to planning horizon both directly and indirectly through general self-efficacy, while reflected appraisal functions as an important facilitating condition. The study clarifies how identity-based self-systems are translated into temporally extended goals under dual-career pressure. Practically, the results suggest that support programmes for student-athletes should not only strengthen academic role identification but also cultivate reflected appraisal and efficacy-related coping beliefs to promote more sustainable long-term planning.

## 1 Introduction

Student-athletes’ career development has been a persistent focus of scholarly attention, as they must balance academic and athletic roles, which require high psychological resilience to withstand the dual career pressures. This study uses the Social Cognitive Career Theory as the theoretical framework to evaluate how student-athletes’ academic orientation may shape their longer-term future planning, and how their capacity to cope with challenges, while this process is further shaped by reflective evaluative tendencies. Student-athletes represent a unique population facing dual-career challenges that often begin early in adolescence. Unlike many other student groups, student-athletes may already need to integrate two demanding roles—student and athlete—while navigating multiple interests, competing expectations, and developmental pressures. At the same time, student-athletes’ dual careers may be structurally time-limited, as athletic performance peaks are often short, and career decisions may need to be made rapidly when performance declines, injuries occur, or retirement becomes unavoidable. Under such circumstances, the ability to sustain a long-term planning horizon may be especially critical, as it enables student-athletes to connect present academic engagement with future career trajectories beyond sport.

The dual-career pathway of student-athletes is not only demanding but also potentially fragile. For example, Wylleman et al. (2004) point out that athletes usually face multiple transitions across stages, and these transitions may become unstable due to performance pressure, selection processes, or injury, highlighting the potential fragility of dual-career trajectories, and dual careers require student-athletes to integrate sport and education simultaneously, which increases developmental complexity and makes their pathways more sensitive to contextual disruptions (Stambulova and Wylleman, 2019). Usenik and Kranjec (2025) also propose that because dual-career athletes must cope with combined sport, academic, and life requirements, the accumulated burden may lead to chronic overload and reduced capacity to maintain balanced functioning over time.

Career transitions and termination represent high-risk periods in elite sport, where athletes may experience substantial psychological challenges and therefore require targeted support to facilitate sustainable adjustment (Di Rocco et al., 2025). In student-athletes, when the dual careers involve crisis transitions in which heightened demands and limited resources can trigger maladaptive adjustment, underscoring the vulnerability of career pathways under stress (Stambulova, 2017). In all, dual-career development does not automatically protect athletes from post-sport difficulties. In contrast, the stability of the career depends strongly on the availability of structured support systems, policy arrangements, or, as we mentioned here, strong and long-term psychological stability (Robnik et al., 2022).

Under such circumstances, how student-athletes cope with stress, regulate their self-beliefs, and engage in reflective evaluation may play a decisive role in shaping their longer-term planning and career direction. Importantly, prior discussions in student-athlete development consistently suggest that many student-athletes may prioritize athletic performance at the expense of sustained academic investment, potentially narrowing their future planning horizons. Moreover, despite the broad influence of Social Cognitive Career Theory in explaining educational and career development, a critical theoretical gap remains in how identity-based self-systems are translated into extended future-oriented goals under conditions of chronic role overload. Although social cognitive career theory has been highly effective in modeling career choice, performance, and domain satisfaction, the psychological processes through which individuals develop temporal extension and long-term planning capacities—particularly in high-demand populations—have not been explicitly articulated. This limitation is especially salient for student-athletes, whose dual-career contexts are characterized by sustained pressure, competing role demands, and constrained time horizons. Therefore, this study adopts a social cognitive career theory-based framework to examine whether general self-efficacy and reflected appraisal help explain how student-athletes with relatively stronger student identity construct a more extended planning horizon. By clarifying these mechanisms, this study aims to advance theoretical understanding of future-oriented goal regulation under chronic role overload and to offer practical implications for educators, sport organizations, and policy makers seeking to support more sustainable and future-oriented dual-career pathways.

**FIGURE 1:**
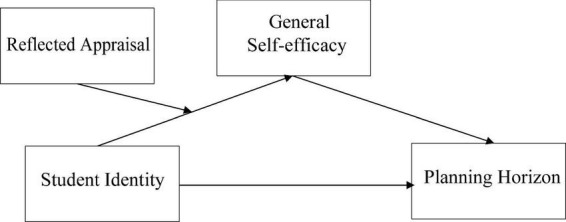
The conceptual model in the study.

Social Cognitive Career Theory is a social-cognitive framework developed to explain how individuals form academic and vocational interests, make educational and career choices, and attain performance and persistence outcomes through key cognitive mechanisms and contextual conditions (Lent et al., 1994). Central to social cognitive career theory are self-efficacy beliefs, outcome expectations, and goal processes, which jointly shape individuals’ motivation, decision-making, and sustained engagement in educational and career domains (Lent et al., 1994, 2000). Over the decades, social cognitive career theory has further expanded beyond its original models, from interest to choice then performance, to include additional formulations addressing domain satisfaction/well-being and career self-management, thereby offering a broader account of how people regulate their development through goal-directed planning across the life span (Lent and Brown, 2006, 2013; Brown and Lent, 2019). This study adopts social cognitive career theory as its overarching theoretical framework because the model under investigation concerns a self-regulatory developmental process in which self-related cognitions shape future-oriented goals. The theoretical framework provides an established, mechanism-based explanation linking self-beliefs to goal formation and adaptive planning in individuals’ careers (Lent et al., 1994; Brown and Lent, 2019). Within this framework, student identity is positioned as a self-system or role-based identity factor that influences how individuals interpret learning experiences and commit to educationally relevant goals, thereby shaping subsequent motivational processes. General self-efficacy is conceptualized as a core efficacy belief that reflects perceived capability for coping and effective functioning, which in social cognitive career theory operates as a central driver of goal setting, persistence, and self-management behavior (Lent et al., 1994; Lent and Brown, 2013). Planning horizon is mapped onto the theory’s goal-related and self-management components, representing an extended future-oriented goal structure through which individuals organize current actions in relation to longer-term trajectories (Lent and Brown, 2013; Brown and Lent, 2019). Finally, reflected appraisal is theorized as a self-regulatory interpretive condition that shapes how identity experiences are cognitively processed into efficacy beliefs and goal construction, which is conceptually consistent with social cognitive career theory’s emphasis on the interpretation of learning experiences and the role of psychological conditions in strengthening or weakening efficacy-based pathways (Lent et al., 2000; Lent and Brown, 2013). The theoretical framework and the model of research is shows as Figure 1.

Student identity among student-athletes can be understood as a role-specific self-concept reflecting the extent to which individuals internalize the student role as central to the self within a dual-career context (Reitzes and Burke, 1980; Lally and Kerr, 2005). Student identity for student-athletes represents more than a formal institutional status, as it captures whether academic participation is perceived as a meaningful life domain rather than a peripheral obligation shaped by athletic schedules (Steele et al., 2020). Within the student-athlete, identity has been widely treated as a foundational construct through which individuals interpret dual career, manage competing role expectations, and construct future directions across academic and sport (Steele et al., 2020). Research suggests that academic identity is generally associated with more adaptive educational outcomes, whereas an overly dominant athletic identity may coincide with less favorable academic indicators. Yukhymenko-Lescroart (2022) reported that academic identity positively predicted academic outcomes, whereas athletic identity showed a negative association. Moreover, student identity may shape future academic decision-making beyond short-term performance, as Foster and Huml (2017) indicated that student/athletic identity relates to academic major selection among intercollegiate student-athletes.

Taken together, student identity is a self-regulatory anchor that stabilizes goal commitment under competing role demands (Lally and Kerr, 2005; Steele et al., 2020; Yukhymenko-Lescroart, 2022). It is important for student-athletes because it provides a stable psychological anchor for academic persistence, supports engagement with long-term academic goals under pressure, and may buffer against the tendency for sport-related demands to override academic self-regulation in everyday life. Also, student identity strengthens the self-system and identity-based regulation, encouraging student-athletes to organize their dual-role, high-pressure lives, set long-term targets, and manage their career considerations.

General self-efficacy is commonly defined as an individual’s broad and relatively stable belief in one’s competence to cope with a wide range of stressful or challenging demands (Luszczynska et al., 2005). It distinguishes from domain-specific self-efficacy that is constrained to particular tasks or contexts. This construct is conceptually grounded in Bandura’s self-efficacy theory, which emphasizes that efficacy beliefs shape how people think, feel, and act when facing difficulties, thereby influencing persistence and self-regulation in goal pursuit (Bandura, 1997).

General self-efficacy is particularly relevant for planning horizon because it reflects perceived capacity to sustain effort across time, not merely across tasks. General self-efficacy offers a theoretically appropriate construct, as it represents a global belief in one’s competence to cope with a broad range of challenging demands (Luszczynska et al., 2005). Also, in dual-career contexts characterized by chronic uncertainty and cross-domain demands, domain-specific efficacy beliefs may be insufficient to sustain extended future planning. The General Self-Efficacy Scale was originally developed to predict coping with daily hassles and adaptation to stressful life events, more relevance to chronic demands and uncertainty (Schwarzer and Jerusalem, 1995). In addition, research suggests that generalized self-efficacy functions as a meaningful personal resource linked to adaptive outcomes across achievement-related contexts (Judge and Bono, 2001). Therefore, this study considered general self-efficacy as an observed coping resource that may help explain how student-athletes translate their academic self-definition into future-oriented planning within a dual-career environment.

Planning horizon refers to the temporal range or extension of individuals’ planning, indicating how far into the future they typically anticipate, set goals, and organize actions in a structured manner (Kooij et al., 2018). This construct is closely aligned with the broader literature on future time perspective, which highlights that individuals differ in how they perceive the future as open or limited and how such time perceptions shape goal structures, preferences, and self-regulatory behavior (Liao and Carstensen, 2018). Planning horizon can be understood as a concept of action of future-oriented cognition, reflecting whether individuals adopt short-term planning that emphasizes immediate demands or long-term planning that prioritizes delayed outcomes and sustained investment. For student-athletes, planning horizon is particularly important because dual-career development requires continuous decisions about training commitments, academic progress, and post-sport transitions, which often involve uncertainty and competing time demands. A limited planning horizon may increase the likelihood that academic decisions become reactive and short-term, whereas an extended planning horizon may support proactive engagement with academic goals and long-term career preparation within and beyond sport.

Future planning is not a passive preference but is part of a goal-directed self-regulatory process through which individuals translate personal beliefs into career actions. Social cognitive career theory emphasizes that self-efficacy beliefs shape goal formation, persistence, and the pursuit of long-term career pathways (Lent et al., 1994). From a career self-management perspective, proactive career actions are largely driven by self-regulatory goals and the cognitive resources supporting them (Lent and Brown, 2013; Hirschi et al., 2021). In this regard, planning horizon can be interpreted as a future-oriented goal structure that reflects one’s capacity to coordinate present actions with longer-term aspirations. Hence, planning horizon is closely related to individuals’ time perspective, as future time perspective and the consideration of future consequences have been consistently linked to future motivation and sustained self-regulation (Bembenutty and Karabenick, 2004; Kooij et al., 2018). Consistent with this view, individuals with stronger general self-efficacy tend to demonstrate higher levels of future planning and consideration of future consequences, suggesting that broad efficacy beliefs may support future cognition and behavior (Azizli et al., 2015). Furthermore, general self-efficacy is tied to a sense of agency, as self-efficacy reflects beliefs about one’s capacity to exert control and influence outcomes. It fosters a stronger conviction that one can actively shape one’s future (Bandura, 1997; Feldman, 2017). In addition, higher self-efficacy has been associated with more adaptive self-regulation patterns, including greater capacity to delay immediate gratification in order to invest in longer-term goals, which supports the development of planning horizon over time (Bembenutty and Karabenick, 2004; Kim et al., 2012). Research has shown that future time perspective is positively associated with academic delay of gratification (Bembenutty and Karabenick, 2004), whereas perceived scarcity or stress may undermine delay of gratification partly through weakened self-efficacy (Yu et al., 2023). These studies support that efficacy-related coping beliefs are closely tied to present–future trade-offs in self-regulated goal pursuit. Conversely, when general self-efficacy is low, individuals may be more likely to focus on immediate pressures and short-term relief strategies, especially under perceived scarcity or stress, which can undermine delay of gratification and narrow future planning (Yu et al., 2023). Therefore, student-athletes with higher general self-efficacy are expected to exhibit stronger planning horizons, whereas those with lower general self-efficacy may show more constrained planning due to heightened present-focused anxiety and coping demands. In the research framework, students’ long term career development or future planning is conceptualized as an agentic and goal-oriented process. Compared to the general temporal preference, planning horizon corresponds to the goal-setting and self-management component of social cognitive career theory. Self-related beliefs and identity-relevant learning experiences shape self-efficacy beliefs, which in turn influence goal formation and future-directed behavior (Lent et al., 1994). In student-athletes, student identity may function as a stagenal role-based self-definition that enhances commitment to educational goals and shapes how academic improvement processes are interpreted in everyday dual-career contexts (Lally and Kerr, 2005; Steele et al., 2020). Such identity processes are particularly relevant for student-athletes because navigating between sport and academics often requires proactive engagement with future planning and transition preparation, rather than relying on short-term, reactive decision-making (Lally and Kerr, 2005). Thus, student identity may be directly linked to a long-term planning horizon, as internalizing the student role can provide a stable psychological status for organizing present academic actions around longer-term educational career trajectories (Steele et al., 2020). Niehues et al. (2025) have also reinforced the importance of identity in dual-career roles, showing that identity-related variables remain central to student-athletes’ motivation and developmental experiences across various stages.

At the same time, social cognitive career theory points out self-efficacy as a core mechanism through which individuals develop persistence, regulate effort, and maintain goal commitment under uncertainty (Bandura, 1997; Lent et al., 1994). General self-efficacy is particularly suitable for student-athletes because it reflects a broad belief in one’s capacity to cope with diverse challenges, and it has demonstrated strong conceptual and measurement validity across cultural contexts (Luszczynska et al., 2005). Importantly, identity-based resources may strengthen efficacy beliefs by influencing how individuals interpret learning experiences, thereby promoting motivation and engagement. Zhang (2025) therefore suggest that identity-related constructs can be positively associated with self-efficacy and performance-relevant outcomes, and that self-efficacy may operate as a mediating mechanism linking identity to engagement-related outcomes (Liao et al., 2024). Although these studies were not conducted in student-athletes, they provide evidence that identity can foster generalized efficacy beliefs that promote sustained future-oriented planning.

Taken together, the study argues that student identity is positively associated with planning horizon not only as a direct identity-based orientation toward long-term goals, but also as an indirect efficacy-based process in which student identity strengthens general self-efficacy, which, in turn, supports an extended planning horizon. For example, research by Wang et al. (2022) shows that target efficacy beliefs and self-management processes are key levers for adaptive academic and career development. Therefore, student identity may contribute to an extended planning horizon not only directly, but also indirectly through general self-efficacy, because an internalized academic role can strengthen students’ perceived capability to cope with diverse challenges, sustain academic investment, and remain engaged in long-term planning. These arguments thus propose hypotheses 1 and 2 for the study:

Hypothesis 1: Student identity is positively associated with planning horizon among student-athletes, such that higher levels of student identity correspond to higher levels of planning horizon.

And meanwhile:

Hypothesis 2: General self-efficacy mediates the relationship between student identity and planning horizon.

Reflected appraisal can be conceptualized as a deliberative, self-relevant evaluative process through which individuals interpret experiences by integrating cognitive meaning and affective information, rather than relying solely on fast, automatic reactions (Lieberman, 2003). Consistent with social cognition theory that distinguishes reflective judgment from intuitive processing, reflected appraisal emphasizes that reflective processes involve controlled interpretation and meaning construction (Lieberman, 2003). In sport psychology, the intuitive or reflected appraisal distinction has also been used to explain how athletes’ performance shapes self-related affect and motivational responses. It suggests that reflected appraisal is not simply a cognitive style but a mechanism that can influence how experiences are psychologically “processed” and internalized (Vallerand, 1987).

For student-athletes, reflected appraisal is important because their career development requires continuous self-regulation across two demanding performance domains, where individuals must repeatedly evaluate feedback, setbacks, role trade-offs, and identity-consistent choices under pressure. In this regard, structured reflective practices may facilitate athletes’ career assistance and adaptive self-management during transitions, supporting the idea that reflective processes contribute to sustaining agency and long-term planning in sport systems (Li et al., 2024). Moreover, student-athletes who focus on identity interventions could be developed by reflective tools and may support identity formation and self-understanding, indicating that reflection can be meaningfully linked to identity integration between athletic and academic (Akiyama et al., 2025).

Within the study framework, self-efficacy beliefs develop through learning experiences and, critically, through how individuals interpret and evaluate those experiences (Bandura, 1977; Lent et al., 1994). Social cognitive career theory argues that adaptive career behavior depends on goal-directed self-regulation and cognitive processing that enable individuals to translate personal beliefs into planning and sustained action over time (Lent and Brown, 2013). From this perspective, reflected appraisal is a theoretical moderator of the relationship between student identity and general self-efficacy. Without reflected appraisal, identity-consistent experiences may fail to accumulate into generalized efficacy beliefs. When reflected appraisal is high, student-athletes who internalize the student role may be more likely to extract efficacy-relevant meaning from academic engagement. Interpreting setbacks as manageable challenges and consolidating academic experiences into broader confidence for coping with diverse demands may strengthen the positive link between student identity and general self-efficacy. In contrast, when reflected appraisal is low, identity-consistent experiences may be less effectively translated into efficacy beliefs because daily demands are processed more reactively, weakening the identity-to-efficacy pathway. Consistent with this reasoning, evidence indicates that reflective capacity is associated with efficacy-related outcomes, implying that reflection may contribute to how competence beliefs are built and maintained (Barkhordari-Sharifabad et al., 2025). Therefore, it is expected that reflected appraisal will moderate the strength of the student identity–general self-efficacy association and, consequently, the indirect effect linking student identity to planning horizon through general self-efficacy, and then the hypotheses 3 and 4 could be proposed:

Hypothesis 3: Reflected appraisal moderates the relationship between Student identity and general self-efficacy among student-athletes, such that the positive association between student identity and general self-efficacy is stronger for those with higher reflected appraisal.

Hypothesis 4: Reflected appraisal moderates the first stage of the mediation process. Specifically, higher levels of reflected appraisal interact with student identity, strengthening the mediating effect of student identity on planning horizon.

Despite the broad influence of Social Cognitive Career Theory in explaining academic and career development, existing research has not sufficiently applied the theory to examine student-athletes’ career choice and planning processes in a systematic manner. As a result, the mechanisms through which student-athletes translate identity-related beliefs into future-oriented goals remain underexplored. Student-athletes may already hold a dual identity as both “students” and “athletes” during adolescence, meaning that they must manage multiple interests, competing role expectations, and developmental pressures at an early stage. Under such circumstances, how student-athletes cope with stress, regulate their self-beliefs, and engage in reflective evaluation may play a decisive role in shaping their longer-term planning and career direction. Importantly, prior discussions in student-athlete development consistently suggest that many student-athletes may prioritize athletic performance at the expense of sustained academic investment, potentially narrowing their future planning horizons. Therefore, the present study focuses on student-athletes with relatively stronger student identity and investigates whether general self-efficacy and reflected appraisal contribute to a more extended planning horizon within a social cognitive career theory-based moderated mediation framework. By clarifying these processes, this study aims to offer practical implications for educators, sport organizations, and policy makers in designing targeted support systems that help student-athletes develop more sustainable and future-oriented dual-career pathways.

## 2 Methodology

### 2.1 Participants and procedures

Anchored in social cognitive career theory, this study aims to explore the relationships among student identity, general self-efficacy, planning horizon, and reflected appraisal among student-athletes. The study recruited participants from intercollegiate athletes to collect and analyze data. The study recruited subjects through purposive sampling, including student-athletes from 14 colleges and universities who completed the questionnaire with their coaches’ consent. The purpose of the study and the test procedure were explained before the test was administered, and the questionnaires were completed only after the subjects had understood and signed the informed consent. A total of 414 participants completed the questionnaires. Among them, 17 participants did not complete all items of the questionnaire and were therefore considered invalid. The final valid sample is 397 participants with a response rate of 95.8%. Among the 397 subjects, 56.0% were male, the average age was 20.2 years old, and the average number of years of experience in sports was 10.52 years. In addition, volleyball (23.9%), taekwondo (22.7%), and table tennis (18.8%) were the three largest sports specialties among the student-athletes, and there were also baseball, softball, basketball, and tennis student-athletes completing the questionnaires in this study.

### 2.2 Measurement

Data collection was conducted through questionnaires composed of the following sections: the Academic and Athletic Identity Scale (AAIS) to measure student identity; the Generalized Self-Efficacy Scale to measure the general self-efficacy; the Planning Horizon Measurement to measure the planning horizon; the Self-Perceptions of Competence and Reflected Appraisals of Significant Others scale to measure reflected appraisals; and personal background variables. Besides the Generalized Self-Efficacy Scale translated by Zhang and Schwarzer (1995), which provides a reliable and valid Chinese version, the other scales were not yet available in Chinese. Thus, the translation of the other scales and responses was conducted according to the steps outlined by Brislin (1980). After the translation was completed, scholars in the fields of sport management and sport psychology were invited to assist in reviewing and revising the questionnaire questions to ensure they had strict expert validity and that the Chinese versions conformed to the meanings of the original questions. To ensure the questionnaire’s validity and that the Chinese questions were consistent with the original questionnaire’s meaning, the questionnaire was formally compiled. The following describes the content of the scales used in the questionnaire.

#### 2.2.1 Student identity

The study used the Academic and Athletic Identity Scale (AAIS) designed by Yukhymenko-Lescroart (2014) to measure the student identity of participants. The full scale of the questionnaire used in the literature consisted of two subparts: the academic identity scale and the athletic identity scale. In this study, only the five items of the academic identity scale were used to assess the degree to which student-athletes identify themselves as students. Questions, such as whether participants are satisfied with their academic work, are used to gauge the level of student identity. The scale used a six-point Likert-type scale; student-athletes were asked to rate how true each statement was for them, using a six-point Likert scale (1 = not central to my sense of self; 6 = very central to my sense of self), with higher scores indicating a more serious with their identities as students. Scoring is done by adding each question’s scores and averaging them. In this study, the Cronbach’s α of this scale was 0.90.

#### 2.2.2 General self-efficacy

The study adopted the Chinese version of the Generalized Self-Efficacy Scale translated by Zhang and Schwarzer (1995). The scale was originally developed by Schwarzer and Jerusalem (1995) with 10 questions, using the four-point Likert scale, where 1 indicates not at all true and 4 indicates exactly true. The scale includes questions like “If someone opposes me, I can find the means and ways to get what I want” and “I can remain calm when facing difficulties because I can rely on my coping abilities” to evaluate the general self-efficacy of student-athletes. Scoring is done by adding up the scores of each question and averaging them. The Cronbach’s α of the scale was 0.92 in the study.

#### 2.2.3 Planning horizon

The study adopted the Planning Horizon Measurement developed by Yu and Zhang (2023), comprising four items, to directly assess participants’ planning horizons. Questions such as “When I need to plan, I usually focus on the proximate future (vs. distant future)” and “Compared to the distant future, I usually act to satisfy my needs and concerns related to the proximate future” were used to anticipate the participants’ future or career planning characteristics. Participants were asked to respond to each statement using a seven-point Likert scale (1 = strongly disagree; 7 = strongly agree). Planning horizon was assessed by averaging item scores and then calculating the mean of these scores. Higher values indicate stronger adaptability in future planning and career considerations. The Cronbach’s α of the scale was 0.86.

#### 2.2.4 Reflected appraisal

This study adopted the six-item Self-Perceptions of Competence and Reflected Appraisals of Significant Others scale (Amorose, 2003), which comprised two subscales to measure participants’ reflected appraisals of their academic and athletic competence. Questions like “How good does (someone important) think you are at your academics?” and “When it comes to your sport, how much ability does (someone important) think you have?” were used to predict student-athletes’ self-perceptions of competence. Responses were scored on a five-point scale, with higher scores reflecting more positive reflected appraisals. The Cronbach’s α of this scale was 0.83 in the study.

#### 2.2.5 Control variables

Building on prior evidence that student-athletes’ academic and athletic identities are not uniform across demographic groups, gender was included as a control variable (0 = male; 1 = female). Steele et al. (2020) summarized that gender differences have been repeatedly observed in student-athletes’ identification and commitment patterns across academic and athletic domains. More recent empirical work likewise indicates meaningful gender variation in academic identity and related motivational orientations among student-athletes (Niehues et al., 2025), and large-scale evidence further shows gender differences in academic identity and time allocation that may shape students’ engagement in academic versus athletic pursuits (Santos and Sagas, 2023). In addition, developmental progression may shift the salience of student-athletes’ roles and future-oriented considerations. Miller and Kerr (2003) reported that as student-athletes mature across their university careers, identity salience can change and some individuals begin to re-evaluate earlier sport-centered investment relative to academic and post-sport considerations. Related qualitative evidence on career planning among university student-athletes also suggests that later-stage student-athletes often recognize the need to rebalance commitment toward academics and future career preparation (Lally and Kerr, 2005). Accordingly, this study included student-athletes’ age and years of training as control variables to account for differences that may influence identity commitment and subsequent career-planning processes.

### 2.3 Data analysis

This study was conducted through IBM SPSS Statistics 30 for data processing and analysis. Descriptive statistics with correlation coefficients were used to present the sample distribution. In addition, this study used Model 4 of the PROCESS module developed by Hayes (2022) to validate the mediation analysis of the research, by using hierarchical regression analysis with the significance criterion set at α = 0.05. Finally, this study used Model 7 of the PROCESS module developed by Hayes (2022) to analyze the effect of moderated mediation. The above statistical tests were conducted by bootstrapping with 5,000 repetitive samples.

## 3 Results

### 3.1 Descriptive analyses

This study employs descriptive statistics to present the distribution of student-athletes’ student identity, general self-efficacy, planning horizon, and reflected appraisal. The mean and standard deviation for student-athletes’ student identity (*M* = 4.20, SD = 0.82), general self-efficacy (*M* = 3.02, SD = 0.43), planning horizon (*M* = 4.50, SD = 0.88), and reflected appraisal (*M* = 3.12, SD = 0.38) are shown in Table 1. Second, utilizing Pearson’s correlation analysis to examine the relationships between variables, results indicate a significant negative correlation between gender and years of sport-specific training (*r* = −0.20, *p* < 0.001), while gender showed a positive correlation with general self-efficacy (*r* = 0.15, *p* < 0.01). Age was significantly positively correlated with years of sport-specific training (*r* = 0.46, *p* < 0.001). Years of sport-specific training were negatively correlated with general self-efficacy (*r* = −0.14, *p* < 0.01). Student identity showed significant positive correlations with general self-efficacy (*r* = 0.33, *p* < 0.001), planning horizon (*r* = 0.48, *p* < 0.001) and reflected appraisal (*r* = 0.28, *p* < 0.001). General self-efficacy was significantly positively correlated with planning horizon (*r* = 0.27, *p* < 0.001) and reflected appraisal (*r* = 0.14, *p* < 0.001). All results are shown in Table 1.

**TABLE 1:** Results of variable distribution and correlation analysis.

Variables	1	2	3	4	5	6	7
1. Gender	–	–	–	–	–	–	–
2. Age	−0.03	–	–	–	–	–	–
3. Years of training	−0.20^***^	0.46^***^	–	–	–	–	–
4. Student identity	0.06	0.04	−0.06	–	–	–	–
5. General self-efficacy	0.15^**^	0.01	−0.14^**^	0.33^***^	–	–	–
6. Planning horizon	0.01	0.06	0.02	0.48^***^	0.27^***^	–	–
7. Reflected appraisal	−0.04	0.04	−0.05	0.28^***^	0.14^***^	0.05	–
Mean	–	20.26	10.52	4.20	3.02	4.50	3.12
Standard deviation	–	1.77	3.18	0.82	0.43	0.88	0.38

*P < 0.05,

**p < 0.01,

***p < 0.001.

### 3.2 The mediating effect of general self-efficacy

To examine the model’s mediating effect, a mediation analysis was conducted using PROCESS Model 4, with student identity as the independent variable, general self-efficacy as the mediator and planning horizon as the outcome variable, controlling for gender, age, and years of sports training. The mediation effect was determined based on the direct and indirect effects of the model. The study utilized bias-corrected bootstrap confidence intervals (Cis) with 5,000 resampling iterations at a 95% confidence level. If the confidence interval did not contain zero, the mediation effect was considered significant.

Firstly, student identity was a significant positive predictor of general self-efficacy (Coefficient = 0.1638, *p* < 0.001, 95% CI [0.1148, 0.2128]). With respect to the control variables, gender was positively associated with general self-efficacy (Coefficient = 0.0911, *p* < 0.05), whereas years of sports training was negatively associated with general self-efficacy (Coefficient = −0.0186, *p* < 0.05). Furthermore, the direct effect of student identity on planning horizon was significant (Direct effect = 0.48, *p* < 0.001, 95% CI [0.3806, 0.5760], not including zero), and the indirect effect student identity on planning horizon was significant too (indirect effect = 0.04, *p* < 0.001, 95% CI [0.0062, 0.0913], not including zero), therefore support the Hypothesis 1 and 2, suggesting a pattern of partial mediation.

Overall, the findings indicate that student identity was positively associated with planning horizon both directly and indirectly through general self-efficacy. Although student identity continued to exert a significant direct effect on planning horizon, the presence of a significant indirect effect supports the partial mediating role of general self-efficacy. Results of regression analysis testing mediation effect are shown in Table 2.

**TABLE 2:** Results of regression analysis testing mediation effect of general self-efficacy on student identity and planning horizon.

	Coefficient	SE	*t*	LL 95% CI	UL 95% CI
Outcome variable: general self-efficacy, R2 = 0.14, F(4, 392) = 15.50, p < 0.001					
Constant	2.14	0.26	8.32^***^	1.63	2.64
Gender	0.09	0.04	2.16*	0.01	0.17
Age	0.02	0.01	1.28	−0.01	0.04
Year of Training	−0.02	0.01	−2.50*	−0.03	−0.004
Student Identity	0.16	0.02	6.57^***^	0.11	0.21
Outcome variable: planning horizon, R2 = 0.25, F(5, 391) = 30.57, p < 0.001					
Constant	1.40	0.53	2.66^**^	0.37	2.43
Gender	−0.05	0.08	−0.58	−0.20	0.11
Age	0.01	0.02	0.32	−0.04	0.06
Year of Training	0.01	0.01	0.95	−0.01	0.04
Student Identity	0.48	0.05	9.62^***^	0.38	0.58
General Self-efficacy	0.27	0.10	2.80^**^	0.08	0.46
**Direct and indirect effect**		**Effect**	**Boot SE**	**LL 95% CI**	**UL 95% CI**
Direct effect of student identity on planning horizon		0.48	0.05	0.38	0.58
Indirect effect of student identity on planning horizon		0.04	0.02	0.01	0.09
Completely standardized indirect effect		0.04	0.02	0.01	0.09

*P < 0.05,

**p < 0.01,

***p < 0.001.

### 3.3 The moderation effect of reflected appraisal on the relationship between student identity and general self-efficacy

To examine whether reflected appraisal moderated the relationship between student identity and general self-efficacy, a moderation analysis was conducted using PROCESS Model 1, with reflected appraisal as the moderator, and gender, age, and years of training included as covariates. The overall regression model was statistically significant, F(6, 390) = 12.20, *p* < 0.001, accounting for 15.81% of the variance in general self-efficacy (R^2^ = 0.1581). Results indicated a significant interaction between student identity and reflected appraisal (Coefficient = 0.1548, *p* < 0.01, 95% CI [0.0533, 0.2562], not including zero), suggesting that the association between student identity and general self-efficacy varied as a function of reflected appraisal. The inclusion of the interaction term resulted in a significant increase in explained variance (ΔR^2^ = 0.0194, F(1, 390) = 8.9993, *p* < 0.01), indicating that reflected appraisal contributed uniquely to the prediction of general self-efficacy beyond the main effects. Table 3 shows the hierarchical regression analysis of the effect of student identity and reflected appraisal and their interaction on general self-efficacy.

**TABLE 3:** Hierarchical regression analysis of the effect of student identity and reflected appraisal and their interaction on general self-efficacy.

Variables	Coefficient	SE	*T*	95% CI	
				LL	UL
Constants	4.21	0.79	5.32^***^	2.6564	5.7682
Gender	0.08	0.04	1.99*	0.0009	0.1658
Age	0.01	0.01	0.87	−0.0143	0.0371
Year of training	−0.02	0.007	−2.25*	−0.0311	−0.0021
Student identity	−0.33	0.16	−2.01*	−0.6508	−0.0065
Reflected appraisal	−0.63	0.23	−2.68^**^	−1.0881	−0.1673
Student identity* Reflected appraisal	0.15	0.05	2.99^**^	0.0533	0.2562
+1 SD Reflected appraisal	0.21	0.03	6.70^***^	0.1501	0.2749
Mean Reflected appraisal	0.15	0.03	5.98^***^	0.1032	0.2044
−1 SD Reflected appraisal	0.10	0.03	2.89^**^	0.0304	0.1598

*P < 0.05,

**p < 0.01,

***p < 0.001; CI, confidence interval; LL, lower limit; UL, upper limit.

**FIGURE 2:**
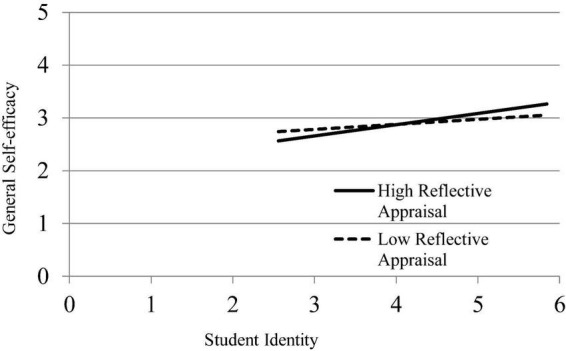
Results of regression interaction of student identity and reflected appraisal.

To further interpret the interaction, conditional effects of student identity on general self-efficacy were examined at low, moderate, and high levels of reflected appraisal. Simple slope analyses revealed that student identity was positively associated with general self-efficacy at all three levels of reflected appraisal; however, the magnitude of this association increased as reflected appraisal increased. Specifically, when reflected appraisal was low, student identity remained a significant predictor of general self-efficacy (Coefficient = 0.0951, *p* < 0.01, 95% CI [0.0304, 0.1598]). This effect was stronger at moderate levels of reflected appraisal (Coefficient = 0.1538, *p* < 0.001, 95% CI [0.1032, 0.2044]) and strongest at high levels of reflected appraisal (Coefficient = 0.2125, *p* < 0.001, 95% CI [0.1501, 0.2749]). These findings indicate that reflected appraisal amplifies the positive relationship between student identity and general self-efficacy, such that student identity is more strongly translated into efficacy beliefs among student-athletes who engage in higher levels of reflective evaluation. In general, the Hypothesis 3 is supported. Figure 2 shows the results of the regression interaction of student identity and reflected appraisal.

### 3.4 The moderated mediation model: the conditional indirect effect of student identity on planning horizon via general self-efficacy

To test the moderated mediation model, we employed the PROCESS macro Model 7 (5,000 bootstraps; Hayes, 2022) to examine the Hypothesis. Before analysis, the research team mean-centered the above variables to mitigate multicollinearity caused by interaction effects. The results showed that the moderated mediation effect was significant, with a moderated mediation index (MMI) of.0414 and a 95% confidence interval (CI) = [0.0020, 0.0946] that did not include zero, indicating the significance of the difference (Hayes, 2022). Thus, the first part of Hypothesis 4 is supported; reflected appraisal moderates the first stage of the mediation among student identity, general self-efficacy, and planning horizon of student-athletes.

Furthermore, conditional indirect effects of student identity on planning horizon via general self-efficacy were examined at different levels of reflected appraisal. At low levels of reflected appraisal, the indirect effect was not significant. In contrast, the indirect effect was significant at moderate levels of reflected appraisal (Coefficient = 0.0411, BootLLCI = 0.0055, BootULCI = 0.0906, not including zero) and at high levels of reflected appraisal (Coefficient = 0.0568, BootLLCI = 0.0083, BootULCI = 0.1160, not including zero), indicating that the indirect effect of student identity on planning horizon through general self-efficacy varied as a function of reflected appraisal. This concludes the model with confirmation that Hypothesis 4 is supported. Table 4 shows the results for the moderated mediation effect.

**TABLE 4:** Bootstrapped results for the moderated mediation effect.

Moderator		Effect (B)	Boot SE	95% CI
Reflected appraisal	−1 SD	0.0254	0.0191	[−0.0011, 0.0712]
	Mean	0.0411	0.0221	[0.0055, 0.0906]
	+1 SD	0.0568	0.0278	[0.0083, 0.1160]
Index of moderated mediation: 0.0414 (Boot SE = 0.0239, 95% CI [0.0020, 0.0946])				

PROCESS macro model 7, 5000 bootstraps.

## 4 Discussion

The present study first examined the descriptive characteristics and bivariate relationships among student identity, general self-efficacy, planning horizon, and reflected appraisal in a sample of student-athletes.

Firstly, student identity was positively associated with general self-efficacy, planning horizon, and reflected appraisal. The positive association between student identity and general self-efficacy is consistent with prior research suggesting that a clear and salient role identity can function as a psychological resource, enhancing individuals’ sense of competence and control (Eccles, 2009; Oyserman et al., 2012). From a social-cognitive perspective, identity serves as a cognitive schema that guides goal-directed behavior, and individuals who strongly identify with their student role are more likely to internalize academic goals, thereby reinforcing their belief in their own capabilities (Bandura, 1997; Markus and Wurf, 1987).

Furthermore, student identity was strongly correlated with planning horizon, indicating that individuals with a stronger orientation toward their student role tend to adopt a more future-oriented perspective. This finding aligns with research on possible selves and future self-continuity, which suggests that identity-relevant domains are closely linked to future planning and goal setting (Oyserman and James, 2011; Hershfield, 2011). When student-athletes perceive their academic identity as central, they may be more likely to consider long-term career development beyond sport, thus extending their planning horizon. This is particularly important in dual-career contexts, where the ability to envision and prepare for post-sport transitions has been identified as a critical adaptive capacity (Stambulova and Wylleman, 2019).

The positive association between student identity and reflected appraisal also provides important insight into the interpersonal dimension of identity construction. The finding suggests that student-athletes with a stronger student identity may also perceive greater social recognition of this role from peers, coaches, and teachers. Such social feedback may reinforce identity salience and consolidate self-related beliefs, thereby supporting adaptive functioning.

General self-efficacy was positively associated with planning horizon. This finding aligns with social cognitive theory, which posits that self-efficacy is a central determinant of individuals’ goal setting, persistence, and future-oriented behavior (Bandura, 1997). Individuals with higher self-efficacy are more likely to set challenging long-term goals and to maintain commitment in the face of obstacles, thereby extending their temporal perspective (Luszczynska et al., 2005).

In addition, general self-efficacy was positively correlated with reflected appraisal, suggesting that social feedback and perceived evaluation from others may contribute to the development of personal efficacy beliefs. This is consistent with the notion that verbal persuasion and social validation are important sources of self-efficacy (Bandura, 1997). When student-athletes perceive that significant others hold positive expectations of them, such appraisals may strengthen their confidence in their own abilities, thereby enhancing their psychological resources.

The relationships among the control variables also warrant consideration. Gender was positively associated with general self-efficacy, while years of sport-specific training showed a negative association with self-efficacy. The latter finding may appear counterintuitive, but it is consistent with research suggesting that prolonged involvement in competitive sport can expose athletes to repeated performance pressures and evaluative stress, which may undermine confidence over time, particularly when performance expectations are high (Gustafsson et al., 2016; Madigan and Nicholls, 2017). Similarly, the positive correlation between age and years of training reflects a typical developmental trajectory in sport participation, and supports the inclusion of these variables as controls.

### 4.1 The mediating role of general self-efficacy

The present findings indicated that student identity was positively associated with planning horizon both directly and indirectly through general self-efficacy, suggesting a pattern of partial mediation. Within the framework of social cognitive career theory, this pattern can be interpreted as evidence that self-efficacy functions as a central cognitive mechanism through which personal self-systems are translated into future-oriented career processes (Lent et al., 1994, 2002). Social cognitive career theory conceptualizes career development as a dynamic system in which self-efficacy, outcome expectations, and personal goals jointly guide individuals’ interests, choices, and performance (Lent et al., 1994, 2002; Wang et al., 2022). In particular, self-efficacy beliefs represent individuals’ perceived capability to perform specific actions, and these beliefs play a decisive role in shaping goal selection, persistence, and engagement in long-term developmental pathways (Bandura, 1997; Lent et al., 1994, 2002). Accordingly, the positive association between student identity and general self-efficacy suggests that internalizing the student role may strengthen student-athletes’ perceived competence in managing academic and career-related demands, thereby facilitating the development of efficacy beliefs that are relevant to long-term planning (Lent et al., 1994, 2002). This interpretation is further supported by social cognitive theory, which indicates that self-efficacy beliefs directly influence individuals’ motivation, effort, and persistence, particularly in contexts requiring sustained engagement and self-regulation (Bandura, 1997; Schunk, 2020; Zimmerman, 2000), thereby providing a theoretical explanation for the significant student identity to general self-efficacy effect observed in the study.

The significant indirect effect further aligns with social cognitive career theory’s assumption that self-efficacy operates as a proximal determinant of goal-directed behavior (Lent et al., 1994, 1996). Within the social cognitive career theory framework, individuals tend to establish goals that are consistent with their perceived capabilities, and these goals function as self-regulatory mechanisms that organize behavior over time (Lent et al., 1994, 1996). Empirical and theoretical work consistently demonstrates that self-efficacy and outcome expectations jointly influence goal formation, which subsequently guides career-related actions and persistence (Lent et al., 1994, 2002; Segal et al., 2002). Social cognitive career theory emphasizes that individuals with higher self-efficacy are more likely to set challenging goals, sustain effort under adversity, and maintain engagement despite barriers, thereby facilitating long-term career development processes (Bandura, 1997; Lent et al., 1994; Lent, 2021). Extensive empirical evidence further indicates that self-efficacy is a strong predictor of persistence, performance, and self-regulated behavior across educational and occupational contexts (Zimmerman, 2000; Honicke and Broadbent, 2016), which helps explain why general self-efficacy was positively associated with planning horizon in the findings. In this regard, the present findings suggest that general self-efficacy enables student-athletes to translate identity-based beliefs into future-oriented planning, as individuals who believe they can manage complex demands are more likely to engage in extended goal-setting and long-term planning behaviors (Lent et al., 1994, 2002). This mechanism is consistent with self-regulation, which posits that efficacy beliefs influence individuals’ ability to coordinate present actions with future goals, thereby extending their temporal planning capacity (Bandura, 1997; Zimmerman, 2000).

Importantly, the persistence of a significant direct effect indicates that student identity influences planning horizon not only relying on self-efficacy (Lent et al., 2002; Lent, 2021). This finding is consistent with our theory framework’s broader proposition that career-related behavior is shaped not only by cognitive evaluations of capability but also by person inputs, values, and contextual influences (Lent et al., 2002; Lent, 2021). Personal factors, including identity-related constructs, may exert both direct and indirect effects on career outcomes through multiple pathways (Lent et al., 2002). The significant direct effect observed in the present study therefore suggests that student identity may function as a value-based or meaning-related system that directly guides individuals’ future orientation, independent of efficacy beliefs. Thus, student identity may contribute to planning horizon not only by enhancing efficacy beliefs, but also by shaping the perceived meaning and value of academic engagement, which can independently motivate future-oriented behavior (Lent et al., 1994, 2002).

Taken together, the significant indirect effect observed in this study further supports the mediating role of self-efficacy as a key explanatory mechanism linking personal beliefs to goal-directed behavior, as consistently proposed within social cognitive career theory (Lent et al., 1994, 2002). At the same time, the observed partial mediation suggests that the translation of identity into long-term planning cannot be fully explained by efficacy beliefs alone, indicating that additional self-regulatory or contextual processes may be involved (Lent et al., 2002; Lent, 2021). This highlights the need to further elaborate the mechanisms through which identity-based self-systems are converted into temporally extended career goals, particularly in populations characterized by multiple role demands.

### 4.2 The moderating role of reflected appraisal

The present findings indicated that reflected appraisal significantly moderated the relationship between student identity and general self-efficacy. Within the study’s theory framework, this pattern can be interpreted as evidence that reflective processes function as a self-regulatory mechanism that facilitates the translation of identity-related beliefs into efficacy judgments. Social cognitive career theory is grounded in broader social cognitive theory, which emphasizes that individuals are not passive recipients of experiences, but actively interpret and evaluate their actions through self-reflection and self-regulation processes (Bandura, 2001). In this regard, reflected appraisal may enhance individuals’ capacity to monitor their performance, interpret feedback, and adjust their self-beliefs accordingly, thereby strengthening the linkage between identity and perceived capability.

The significant interaction effect further suggests that reflected appraisal strengthens, rather than determines, the association between student identity and self-efficacy, such that identity-based beliefs are more strongly translated into efficacy judgments at higher levels of reflective evaluation. This finding is consistent with theoretical perspectives on self-regulated learning, which propose that self-reflection is a critical phase in the self-regulatory cycle, enabling individuals to evaluate their performance, attribute outcomes, and update their efficacy beliefs (Zimmerman, 2002). Individuals who engage in higher levels of reflection are more likely to transform experiences into adaptive self-knowledge, which in turn enhances their perceived competence and confidence (Schön, 1983; Grant et al., 2002). Accordingly, the present findings suggest that reflected appraisal may act as a cognitive filter through which identity-related experiences are interpreted, thereby determining whether such experiences contribute to the development of self-efficacy.

The simple slope analysis provides further support for this interpretation, showing that the effect of student identity on general self-efficacy was significant at all levels of reflected appraisal but increased substantially as reflected appraisal increased. This pattern indicates that reflected appraisal amplifies, rather than replaces, the effect of student identity, suggesting a synergistic relationship between identity and self-reflection. From a social cognitive perspective, self-reflection allows individuals to construct coherent self-beliefs by integrating past experiences with current role identities, thereby enhancing their sense of agency and perceived control (Bandura, 2001; Morin, 2006). As a result, student-athletes who engage in higher levels of reflected appraisal may be better able to interpret their academic experiences as evidence of competence, which strengthens the identity–efficacy linkage.

Conversely, when reflected appraisal is low, individuals may have difficulty interpreting their experiences in a constructive manner, leading to weaker associations between identity and self-efficacy. Prior research has shown that limited reflective capacity may hinder individuals’ ability to derive learning from experience, thereby constraining the development of adaptive self-beliefs (Kember et al., 2000). In such cases, even if individuals possess a strong student identity, the absence of reflective processing may reduce the extent to which this identity translates into perceived competence. This finding highlights the importance of reflected appraisal as a boundary condition in the development of self-efficacy, suggesting that identity-based beliefs require active cognitive processing in order to influence self-evaluative judgments.

### 4.3 Moderated mediation mechanism of reflected appraisal

The moderated mediation findings suggest that the efficacy-based pathway linking student identity to planning horizon is conditional on the level of reflected appraisal. Student-athletes’ self-efficacy is conceptualized as a proximal cognitive mechanism through which personal attributes are translated into goal-related behaviors and future-oriented actions (Lent et al., 1994; Lent and Brown, 2013). In this sense, planning horizon can be understood as a form of career self-management outcome, reflecting individuals’ capacity to extend their temporal perspective and organize long-term developmental goals under multiple role demands (Lent and Brown, 2013; Brown and Lent, 2019). The research findings indicate that this identity–efficacy–planning process does not operate uniformly but depends on individuals’ reflective capacity to process and integrate role-related experiences.

The pattern of conditional indirect effects provides further insight into this psychological mechanism. When reflected appraisal was low, the indirect effect of student identity on planning horizon through general self-efficacy was not significant, suggesting that a strong student identity alone may not consistently translate into broader efficacy beliefs and extended future planning when reflective evaluative capacity is limited. Under such conditions, student-athletes may hold a salient student identity but lack the cognitive resources required to critically appraise experiences, integrate feedback, and generalize identity-relevant information into stable beliefs about their capability to manage future demands. From a social cognitive career theory perspective, self-efficacy is shaped not only by performance experiences but also by how such experiences are cognitively interpreted and organized (Lent et al., 1994, 2017). Without sufficient reflective processing, identity-consistent experiences may remain fragmented, thereby weakening their contribution to efficacy formation and subsequent goal regulation.

In contrast, at moderate and high levels of reflected appraisal, the indirect effect became significant and progressively stronger. This pattern indicates that reflected appraisal functions as a key enabling condition that allows student-athletes to transform identity-based self-definitions into general self-efficacy, which in turn supports longer planning horizons. For student-athletes who actively engage in reflective evaluation, academic role identification may be more effectively integrated into broader self-regulatory beliefs, facilitating sustained effort, adaptive coping, and investment in long-term goals. This finding aligns with the career self-management extension of social cognitive career theory, which emphasizes that individuals actively regulate their development through cognitive processes that link self-beliefs, environmental feedback, and goal-directed behaviors (Lent and Brown, 2013; Brown and Lent, 2019). Reflected appraisal, in this context, may serve as a mechanism that enhances the diagnostic value of learning experiences, allowing identity-relevant information to be translated into efficacy beliefs that guide future-oriented planning.

Taken together, these findings suggest that reflected appraisal not only strengthens the immediate link between student identity and general self-efficacy but also determines whether this psychological pathway ultimately contributes to extended future-oriented planning. This conditional process helps explain why some student-athletes are able to leverage their student identity into long-term career planning, whereas others remain focused on more immediate demands despite holding similar identity orientations. Such differentiation is consistent with prior social cognitive career theory research showing that the strength of cognitive pathways may vary depending on contextual and personal conditions that influence how individuals process experience and regulate behavior (Sheu et al., 2010; Brown and Lent, 2019).

### 4.4 Practical implications for supporting student-athletes’ academic identity and reflective capacity

Building upon these findings, several practical directions may be considered to strengthen the identity–reflection–efficacy pathway identified in this study. First, institutional policies that enhance academic integration may contribute to the internalization of the student role among student-athletes. Most studies on student-athletes indicate that a sense of belonging, expectations, and support within the academic environment can promote the internalization of the academic role—corresponding to what the present study conceptualizes as student identity—while reducing the risk of a narrowly defined “athlete-only” identity (Steele et al., 2020). From a policy perspective, the design of academic support services—such as advising, tutoring, and learning support—in ways that are highly accessible, routinely available, and non-punitive has been considered beneficial for strengthening academic engagement and academic orientation among student-athletes (West, 2021). In addition, academic identity is positively associated with academic performance, suggesting that institutional efforts aimed at reinforcing academic identity may represent a reasonable and effective developmental strategy (Yukhymenko-Lescroart, 2023). When the system repeatedly reinforces the athletic role as the dominant source of recognition, opportunities for academic exploration and long-term career preparation may become constrained (Foster and Huml, 2017). Therefore, policies that encourage team cultures to openly recognize academic engagement as a legitimate and valued form of achievement may contribute to the internalization of student identity among student-athletes (Steele et al., 2020). When student-athletes are guided to engage in career planning, role transition awareness, and the identification of transferable skills, they may be more likely to perceive the student role as part of their long-term self-development rather than a short-term obligation (Forester et al., 2020). At the same time, programmes involving community engagement or service participation are often used to stimulate broader self-concepts and career imagination among student-athletes (Martin, 2018). Such approaches may serve as upstream policy tools that facilitate the expansion of planning horizons discussed in the study.

Second, reflected appraisal appears to be a skill that can be cultivated rather than assumed. Structured reflective practices, such as guided journaling, post-performance debrief conversations, and goal–review cycles embedded within training routines, have been shown to enhance reflective depth and self-regulatory awareness in sport contexts (Da Silva et al., 2022; Latinjak et al., 2018). Moreover, the design of guided reflection has been implemented in high-performance environments as a means of strengthening self-monitoring and reflective dialogue (Koh and Wang, 2015). Within sport contexts, reflection interventions may be more effective when aligned with the self-regulation cycle—for example, through post-training review, attribution of errors, and the formulation of subsequent performance goals. Experimental and intervention-based research suggests that combining reflection with systematic review processes can enhance goal-directed self-talk and performance-related self-monitoring (Latinjak et al., 2018). Additional studies focusing on academy and developmental athletes further indicate that specific psychological skills tools can also facilitate reflective processes and self-regulatory capacity (McGreary et al., 2024). Within high-performance sport and applied sport psychology, group-based reflection and peer feedback are often considered practical approaches for increasing both the frequency and quality of reflective practice (Neil et al., 2013). For student-athletes, institutionalizing reflective activities—such as short weekly reflection sessions involving guided prompts and peer discussion—may help cultivate more stable habits of reflected appraisal (Downham et al., 2024). Such approaches may strengthen student-athletes’ capacity to interpret academic experiences as efficacy-relevant evidence rather than isolated events.

Finally, the statistical results of the present study indicate that the effect of identity on efficacy becomes stronger when reflected appraisal is higher. Therefore, in terms of policy design, a two-stage approach may be adopted in which student identity is first strengthened and subsequently consolidated through reflective processes. For example, institutions may first normalize academic advising systems and academic goal structures (such as GPA targets or course planning), and then employ guided reflection to help student-athletes interpret their academic engagement as transferable competence and a sense of personal agency. To make the interaction mechanism more explicit, reflective prompts should not merely ask general questions such as “How was today?” but instead direct attention to identity-relevant experiences. For instance, prompts may ask: “Which academic behavior today most reflects your role as a student?” or “How did this experience influence your imagination of future academic or career pathways?” Such prompts may connect reflective processing with identity-relevant evidence, thereby facilitating a more effective translation from identity to efficacy beliefs (Da Silva et al., 2022; Neil et al., 2013). One of the core challenges faced by student-athletes is the tension between multiple roles. Interventions that explicitly address dual-career transitions, role integration, and the identification of transferable skills may therefore be more effective in transforming student identity into a sustainable long-term self-concept. Such programmes may also guide reflective processes toward integration rather than self-doubt, thereby encouraging student-athletes to interpret their experiences as part of a coherent developmental trajectory (Steele et al., 2020; Stambulova et al., 2021). In sum, this logic of combining systemic support with reflective processing is consistent with the dual-career intervention literature, which emphasizes the integration of career planning, life skills development, and identity formation in athlete development programmes (Stambulova et al., 2021).

## 5 Conclusion

This study applied Social Cognitive Career Theory to clarify how student-athletes’ academic orientation is translated into extended future-oriented planning. The findings support a coherent self-regulatory account in which student identity is positively linked to planning horizon both directly and indirectly through general self-efficacy, while reflected appraisal operates as an important boundary condition that strengthens the identity-to-efficacy pathway and, consequently, the conditional indirect effect on planning horizon.

First, student identity showed a positive association with general self-efficacy, indicating that internalizing the student role is accompanied by stronger global coping beliefs. Identity-consistent academic engagement can be interpreted as a context in which efficacy-relevant meaning is accumulated and consolidated, thereby supporting broader confidence to manage challenges across domains. Second, general self-efficacy was positively associated with planning horizon, suggesting that individuals who believe they can cope effectively are more able to sustain goal-directed regulation over time and organize present actions in relation to longer-term aspirations. Together, these results support the central mechanism proposed in this study: efficacy beliefs represent a key psychological route through which identity-based self-systems are linked to extended future-oriented goals.

The mediation pattern was partial rather than complete. Student identity remained directly associated with planning horizon other than the efficacy pathway. This implies that academic role identification may shape future-oriented planning not only through perceived capability but also through identity-based meaning, commitment, and value-related self-regulation that are not fully captured by general self-efficacy. In other words, student identity appears to function as a self-system that can directly orient individuals toward long-term trajectories, while self-efficacy explains an additional—but not exhaustive—portion of this process.

The moderation findings further refine the interpretation of the theory framework. Reflected appraisal significantly strengthened the positive association between student identity and general self-efficacy, indicating that identity does not operate as a “fixed input,” but becomes psychologically influential through interpretive self-regulatory processing. Notably, the identity–efficacy association remained significant even when reflected appraisal was relatively low, yet the magnitude of this association increased as reflected appraisal increased. This pattern suggests an amplification mechanism: reflected appraisal does not determine whether identity matters, but it increases how strongly identity-related experiences are translated into broad efficacy beliefs.

Consistent with this interpretation, the moderated mediation results indicate that the indirect effect of student identity on planning horizon via general self-efficacy became reliable at moderate and high levels of reflected appraisal. Conceptually, this conditional process implies that reflected appraisal is a psychologically meaningful “enabling condition” for temporal extension in high-demand contexts: student identity can be present, but without sufficient reflective evaluation, identity-consistent experiences may be less likely to generalize into broad coping beliefs that support longer-term planning. In contrast, when reflected appraisal is stronger, identity-related beliefs appear more likely to be consolidated into general self-efficacy, which then supports more extended planning horizons. This finding addresses the theoretical gap motivating the present study by offering an explicit social cognitive career theory-related account of how identity-based self-systems are translated into temporally extended goals under chronic role overload.

Regarding the limitations and future research directions, from a theoretical perspective, the study extends social cognitive career theory by specifying reflected appraisal as a boundary condition that shapes whether and how identity-related self-systems are converted into efficacy beliefs that support temporal extension in planning. From a practical perspective, the results suggest that support for student-athletes should not only promote academic role identification but also cultivate reflective evaluation skills, because reflection strengthens the conversion of identity into broad coping beliefs and future-oriented planning. A key limitation is that the study relied on cross-sectional self-report data; therefore, the temporal ordering implied by the model cannot be confirmed causally, and future longitudinal or intervention-based research is needed to test dynamic changes in identity, reflected appraisal, efficacy formation, and planning horizon over time.

## Data Availability

The datasets presented in this article are not readily available because data may only be available upon reasonable request to the authors, rather than being openly accessible. Requests to access the datasets should be directed to hsinchuan@ntu.edu.tw.
